# Limited Contribution of IL-36 versus IL-1 and TNF Pathways in Host Response to Mycobacterial Infection

**DOI:** 10.1371/journal.pone.0126058

**Published:** 2015-05-07

**Authors:** Noria Segueni, Solenne Vigne, Gaby Palmer, Marie-Laure Bourigault, Maria L. Olleros, Dominique Vesin, Irene Garcia, Bernhard Ryffel, Valérie F. J. Quesniaux, Cem Gabay

**Affiliations:** 1 CNRS, UMR7355, Orleans, France; 2 University of Orleans, Experimental and Molecular Immunology and Neurogenetics, Orleans, France; 3 Division of Rheumatology, Department of Internal Medicine Specialties, University Hospitals of Geneva, Geneva, Switzerland; 4 Department of Pathology and Immunology, University of Geneva Medical School, Geneva, Switzerland; 5 Division of Immunology, Institute of Infectious Disease and Molecular Medicine, Health Sciences Faculty, University of Cape Town, Cape Town, South Africa; Institut Pasteur, FRANCE

## Abstract

IL-36 cytokines are members of the IL-1 family of cytokines that stimulate dendritic cells and T cells leading to enhanced T helper 1 responses *in vitro* and *in vivo*; however, their role in host defense has not been fully addressed thus far. The objective of this study was to examine the role of IL-36R signaling in the control of mycobacterial infection, using models of systemic attenuated *M*. *bovis* BCG infection and virulent aerogenic *M*. *tuberculosis* infection. IL-36γ expression was increased in the lung of *M*. *bovis* BCG infected mice. However, IL-36R deficient mice infected with *M*. *bovis* BCG showed similar survival and control of the infection as compared to wild-type mice, although their lung pathology and CXCL1 response were transiently different. While highly susceptible TNF-α deficient mice succumbed with overwhelming *M*. *tuberculosis* infection, and IL-1RI deficient mice showed intermediate susceptibility, IL-36R-deficient mice controlled the infection, with bacterial burden, lung inflammation and pathology, similar to wild-type controls. Therefore, IL-36R signaling has only limited influence in the control of mycobacterial infection.

## Introduction

IL-36 cytokines belong to the IL-1 family of cytokines and include three agonists IL-36α, IL-36β, and IL-36γ that bind to IL-36R leading to the recruitment of IL-1RAcP, the common co-receptor for IL-1 and IL-33, and to activation of similar intracellular pathways as other IL-1 cytokine family members [1]. IL-36 receptor antagonist (IL-36Ra) binds also to IL-36R but does not induce any signaling and acts as a natural IL-36 inhibitor, thus sharing similar to IL-1Ra for IL-1. We and others have recently shown that IL-36R is expressed by human and mouse dendritic cells (DC) and that IL-36 stimulates the production of several cytokines by DC, including TNF-α, IL-1, IL-12, and IL-23, as well as the maturation of DC with enhanced expression of MHC class 2 and co-stimulatory molecules [2], [3], [4]. Human T cells do not express IL-36R and fail to respond directly to IL-36 [4], [3]. However, IL-36 stimulation of human monocyte-derived DC is able to induce the production of IFN-γ in T cell/monocyte DC allogeneic co-cultures, thus suggesting that IL-36 can promote Th1 responses in human cells [3]. In contrast, IL-36R is expressed by mouse CD4+ T cells, and more specifically by naïve CD4+ T cells. Accordingly, IL-36, but not other IL-1 family cytokines, is able to stimulate the proliferation and IL-2 release by naïve CD4+ T cells. In combination with IL-12, IL-36 markedly stimulated the polarization of naïve T cells into Th1 cells *in vitro* [5], and intradermal injection of IL-36 in an immunization protocol was able to induce Th1 responses *in vivo* [2]. Taken together, these findings suggest that IL-36 could play a critical role in host defense against pathogens, particularly against intracellular microorganisms controlled by Th1 responses. Consistent with this hypothesis, we recently observed that production of IFN-γ, TNF-α, IL-6 and nitric oxide (NO) was decreased in *ex vivo* stimulated splenocytes of IL-36R deficient mice infected by *M*. *bovis* BCG as compared to wild-type mice [5].

It is generally accepted that less than 10% of individuals infected with *Mycobacterium tuberculosis* will develop an active disease, suggesting that the host immune system is usually efficient in the control of mycobacteria, although the infection is not cleared and can remain in a latent form for many years [6], [7]. Immunodepression of the host can favor a reactivation of latent tuberculosis infection. Indeed, coordinated innate and adaptive immune responses are required for efficient control of *M*. *tuberculosis* infection, including T cells, macrophages, and the expression of IFN-γ, TNF-α, IL-1, IL-12, NO, reactive oxygen and reactive nitrogen intermediates [8], [9], [10], [11], [12]. One of the most compelling example regarding the role of the immune system in controlling *M*. *tuberculosis* infection is the reactivation of latent tuberculosis, as well as increased susceptibility to primary tuberculosis infection in patients treated for inflammatory diseases such as rheumatoid arthritis, psoriasis and Crohn’s disease with TNF-α antagonists [13], [14], [15], [16]. In mice, the IL-1R1 pathway seems essential for the control of acute *M*. *tuberculosis* infection, and both IL-1α and IL-1β have been implicated [17], [18], [19], [20], [21], [22], [23], [24]. We therefore addressed the contribution of IL-36 cytokines and of the IL-36R signaling pathway in the control of mycobacterial infection by using IL-36R gene deficient mice in two models, namely *M*. *bovis* BCG systemic infection and *M*. *tuberculosis* aerogenic infection. This question has potential clinical implication in the context of future drugs targeting IL-36R signaling. Indeed, several lines of evidence indicate that IL-36 signaling is involved in the pathogenesis of psoriasis (reviewed in [25]), thus leading to consider the development of therapies blocking IL-36 for this indication.

The results described herein show that while both TNF-α and IL-1R1 pathways were required to control *M*. *tuberculosis* infection, absence of functional IL-36R did not compromise survival and bacterial clearance in *M*. *bovis* BCG and *M*. *tuberculosis* infections.

## Materials and Methods

### Mice

WT C57BL/6 and mice deficient for IL-36R [26], IL-1R1 [27], and TNF-α [28] were housed in the Transgenose Institute animal facility (CNRS UPS44, Orleans). WT C57BL/6 and mice deficient for IL-36R were also housed in the animal facility of the Centre Medical Universitaire, University of Geneva. The generation of IL-36R KO mice was previously described [26]. These mice were crossed for at least 9 generations with C57BL/6 mice. The backcross into the C57BL/6 background was performed by using a marker-assisted selection protocol (MASP) approach, also termed “speed congenics” [29]. The other KO mice were backcrossed at least 7–10 times on C57BL/6 genetic background. For infection with *Mycobacterium bovis* BCG, adult mice (8–12 week old) were kept in a conventional animal facility (Geneva). For *Mycobacterium tuberculosis* infection, adult (8–12 week old) animals were kept in isolators in a biohazard animal unit (Orleans). The infected mice were monitored regularly for clinical status and weighed twice weekly. Water and food were provided ad libitum. All animal experiments on *M*. *bovis* BCG and *M*. *tuberculosis* were approved by the Geneva cantonal authority for animal experimentation (authorization N° 31.1.1005/3202/2) and by the Ethics Committee for Animal Experimentation of CNRS Campus Orleans” (CCO; N° CLE CCO 2012–1001), respectively.

### Intratracheal instillation of lipopolysaccharide (LPS)

200 μg LPS (*Escherichia coli* serotype O55:B5) dissolved in 50 μl sterile 0.9% NaCl was instilled intratracheally using a canula into anesthetized mice. Control mice were instilled intratracheally with 50 μl LPS-free sterile 0.9% NaCl. The mice were euthanized 6 h after instillation and lungs collected for total RNA preparation.

### Bacteria and infection

#### *M*. *bovis* BCG infection

Adult WT and IL-36R KO mice were infected intravenously (i.v) with 5x10^6^ CFU living *M*. *bovis* BCG Pasteur strain 1173 P2 as previously described [30]. Humane endpoints were used during the *M*. *bovis* BCG infection survival study. Mice were observed daily and weighed twice a week. To minimized animal distress, mice presenting signs of distress or bodyweight loss were examined twice a day, and mice showing prostration or body weight loss ≥20% were euthanized by isofluorane anesthesia, exsanguination, and cervical dislocation. Mice were sacrificed 28 or 180 days post-infection. Lung bacterial load was evaluated by plating lung homogenates on 7H11 agar plates and counting the number of colonies.

#### *M*. *tuberculosis* infection

*M*. *tuberculosis* H37Rv (Pasteur) aliquots kept frozen at -80°C were thawed, diluted in sterile saline containing 0.05% Tween 20 and clumping was disrupted by 30 repeated aspirations through a 26 gauge needle (Omnican, Braun, Germany). Pulmonary infection with *M*. *tuberculosis* H37Rv was performed by instillation of 1000–3000 CFU/lung into the nasal cavities (20μl each) under xylazine-ketamine anaesthesia (100mg/kg ketamine, 10mg/kg xylazine, i.p.), and the inoculum size was verified 24 h after infection by determining bacterial load in the lungs. Humane endpoints were used during the *M*.*tuberculosis* infection survival studies. Mice were observed daily and weighed twice a week. To minimize animal distress, mice presenting signs of distress or bodyweight loss were examined twice a day, and mice showing prostration or bodyweight loss >20% were euthanized by cervical dislocation. There was no incident or unexpected mortality in the infected animals.

*M*. *tuberculosis* bacterial loads in the lung of infected mice were evaluated at different time points after infection with *M*. *tuberculosis* H37Rv as described [31]. Organs were weighed and defined aliquots were homogenized in PBS in a Dispomix homogenizer (Medic Tools, Axonlab, Baden-Daettwil, Switzerland). Tenfold serial dilutions of organ homogenates in 0.05% Tween 20 containing 0.9% NaCl were plated in duplicates onto Middlebrook 7H11 (Difco) agar plates containing 10% OADC and incubated at 37°C. Colonies were enumerated at 3 weeks and results are expressed as log_10_ CFU per organ.

### Cytokine determination

Lung homogenates were centrifuged (3 min at 14,500 rpm), the supernatants sterilized by centrifugation through 0.22 mm filter (3 min at 14,500 rpm; Costar-Corning, Badhoevedorp, The Netherlands), immediately frozen on dry ice and stored at -80°C until determination of CXCL1 and IFNγ levels by ELISA (Duoset R&D Systems, Abingdon, UK).

### RNA extraction and RT-qPCR

Total RNA was extracted from lungs of *M*. *bovis* BCG and *M*. *tuberculosis* infected mice, as well as after LPS challenge using TRIzol reagent, according to manufacturer’s instructions, and further purified on RNeasy columns (Qiagen AG, Hombrechtikon, Switzerland). Total RNA (1 μg) was reverse transcribed using SuperScript II Reverse transcriptase (Invitrogen Life Technologies). The mRNA levels for genes of interest were examined by quantitative RT-PCR using SYBR Green PCR Master Mix (Applied Biosystems, Foster City, CA). Values obtained with the SDS 2.2 (Applied Biosystems, Zug, Switzerland) were imported into Microsoft Excel for analyses and gene expression was calculated using the comparative method (2^–ΔCt^) for relative quantification by normalization to *Gapdh* or *L32* gene expression. The primer sequences used for real-time quantitative RT-PCR are described in Table 1.

**Table 1:** Nucleotide sequences of primers used for RT-qPCR.

Primer sequences for RTqPCR		
cDNA	forward primer	reverse primer
Gapdh	5'-acggccgcatcttcttgtgca-3'	5'-aatggcagccctggtgacca-3'
L32	5'-gaaactggcggaaaccca-3'	5'-ggatctggcccttgaacctt-3'
Il36α	5'tagtgggtgtagttctgtagtgtgc-3'	5'gttcgtctcaagagtgtccagatat-3'
Il36β	5'-acaaaaagcctttctgttctatcat-3'	5'-ccatgttggatttacttctcagact-3'
Il36γ	5'-agagtaaccccagtcagcgtg-3'	5'-agggtggtggtacaaatccaa-3'
Il36r	5'-aaacacctagcaaaagcccag-3'	5'-agactgcccgattttcctatg-3'
Tnfα	5'-agttctatggcccagaccct-3'	5'-gtctttgagatccatgccgt-3'
Il1β	5'-tgtgaaatgccaccttttga-3'	5'-gtgctcatgtcctcatcctg-3'
Ifnγ	5'-agacaatcaggccatcagca-3'	5'-tggacctgtgggttgttgac-3'
Cxcl1	5'-actcaagaatggtcgcgagg-3'	5'-gtgccatcagagcagtctgt-3'
Cxcl2	5'-agggcggtcaaaaagtttgc-3'	5'-cgaggcacatcaggtacgat-3'
Il6	5'-tgaacaacgatgatgcacttgcaga-3'	5'-tctgtatctctctgaaggactctggct-3'

### Histopathological analysis

For histological analysis lungs from *M*. *bovis* BCG and *M*. *tuberculosis* infected mice were fixed in 4% phosphate buffered formalin, paraffin-embedded, and 4 μm sections (for *M*. *bovis* BCG) and 3 μm sections (for *M*. *tuberculosis*) stained with haematoxylin and eosin and a modified Ziehl-Neelsen method. The latter involved staining in a prewarmed (60°C) carbol-fuchsin solution for 10 min followed by destaining in 20% sulphuric acid and 90% ethanol before counterstaining with methylene blue. Histopathological analyses were done by quantification of lung tissue and lung free of lesions on tissue sections comprising a lung surface of 45 ± 20 mm^2^ corresponding to one or two lobe sections per animal. Percentage of free alveolar space corresponds to % lung lesion free /lung tissue. Scorings were determined by a pathologist using digital microscopic images acquired by Mirax Digital Slide Scanner and Mirax Viewer (Carl Zeiss).

### In situ hybridization study

*In situ* hybridization was performed at the Histo-Pathology / in situ Hybridisation Core Facility (Faculty of Medicine, Geneva University). A DNA template for riboprobe synthesis was generated from a plasmid containing the cDNA for murine *Il36g*. This template was obtained by PCR using the forward primer mmu-IL36G_SP6 (5’CCG**ATTTAGGTGACACTATAGAA**AACAGGAATGGCTTCATTGG 3’) and the reverse primer mmu-IL36G_T7 (5’CCG**TAATACGACTCACTATAGGG**CGAGAGAGCTGGGCTATTTG 3’). *In vitro* transcription was carried out in a cocktail of 1mM rATP, rCTP, rGTP, 0.65mM rUTP, and 0.35mM digoxigenin-UTP (RNA kit labeling from Roche), 1 ml of ribonuclease inhibitor (40 U/ml MBI Promega), 0.5 mg of DNA template, 0.5 ml of SP6 RNA polymerase (SP6: 20 U/l, New England Biolabs), in a final volume of 20 ml. The reaction mix was incubated at 37°C for 2.5 hours followed by a 15 mn incubation with a DNase I/MgCl2 mix to remove the DNA template (1.6ml of 300 mM MgCl2, 2 ml of DNase I [10 U/l, Roche], 16.4 l of DEPC water). RNA was ethanol precipitated and dissolved in DEPC water to 150 ng/ml. 4um thick sections from paraffine-embedded murine tissues fixed previously in formol were applied on Superfrost slides and loaded onto the Discovery automated slide-processing system (Ventana Medical Systems, Inc). Baking and deparaffinization steps were performed as programmed in the protocol for the RiboMap in situ hybridization reagent system (Ventana Medical Systems, Inc) on the instrument. In situ hybridization protocol was designed based on the standard protocol described in the manufacture's RiboMap application note. The first fixation step was performed using formalin-based RiboPrep reagent for 36 minutes at 37°C. Then, the sections were acid-treated using hydrochloride-based RiboClear reagent for 12 minutes at 37°C. Slides were then processed for protease digestion using Protease 1 reagent (0.5U/ml) for 4 minutes at 37°C and incubated with the IL36g antisense riboprobes (100 ng/slide) in RiboHybe hybridization buffer (Ventana Medical Systems, Inc) for 6 hours at 65°C. After 3 stringency washes using 0.1× RiboWash (Ventana Medical Systems) for 8 minutes each at 75°C, the second fixation step was performed using RiboFix reagent for 32 minutes at 37°C followed by incubation of Alcaline Phosphatase-labeled antidigoxigenin antibody (Roche, dilution 1/500) for 32 minutes at 37°C. The signal was detected automatically using the BlueMap NBT/BCIP substrate detection kit (Ventana Medical Systems, Inc.) for 6 hours at 37°C. Finally, the sections were counterstained with a nuclear fast red equivalent reagent ISH Red (Ventana Medical Systems, Inc) for 4 minutes before coverslipping.

### FACS analysis of infiltrating cells in infected lungs

After perfusion with 0.02% EDTA-PBS and removal, lung tissue was sliced into 1 to 2 mm^3^ pieces and was incubated in RPMI 1640 (Gibco, Paisley, Scotland, UK) containing antibiotics (Penicilline 100 U/ml-Streptomycine 100 μg/ml), 10 mM Hepes (Gibco) and collagenase (150U/ml), DNase (50U/ml; Sigma, St Louis, MO). After 45min of incubation at 37°C, single-cell suspension was obtained by vigorous pipetting and filtering through 100μm and 20μm. Cells were washed three times in RPMI 1640 containing 5% BSA and were then stained according to antibody manufacturer protocols. Rat anti-mouse CD11b-FITC (clone M1/70), Gr1-PECy7 (clone RB6-8C5), were from BD Pharmingen (San Diego, CA). Stained cells were washed twice, fixed with 1% paraformaldehyde (FACS Lysing solution, BD) and analyzed by flow cytometry on a CANTO II analyser (Becton Dickinson). Data were processed with FlowJo software (version 7.6.5 for Windows, FlowJo LLC, Ashland, Oregon).

### Statistical analysis

Statistical significance was determined with Graph Pad Prism (version 5.04 for Windows, GraphPad Software, La Jolla, CA). Differences between multiple *in vivo* groups were analyzed by one-way non-parametric test (Kruskal-Wallis), before comparing individual groups using a Mann-Whitney test. Survival curve were compared using Log-rank (Mantel-Cox) test. Evolution of body weight was compared by using two-way ANOVA for repeated measures, followed by Sidak’s multiple comparison test. Values of *p* ≤ 0.05 were considered significant.

## Results

### IL-36γ expression is enhanced in response to lung inflammation

**Fig 1:**
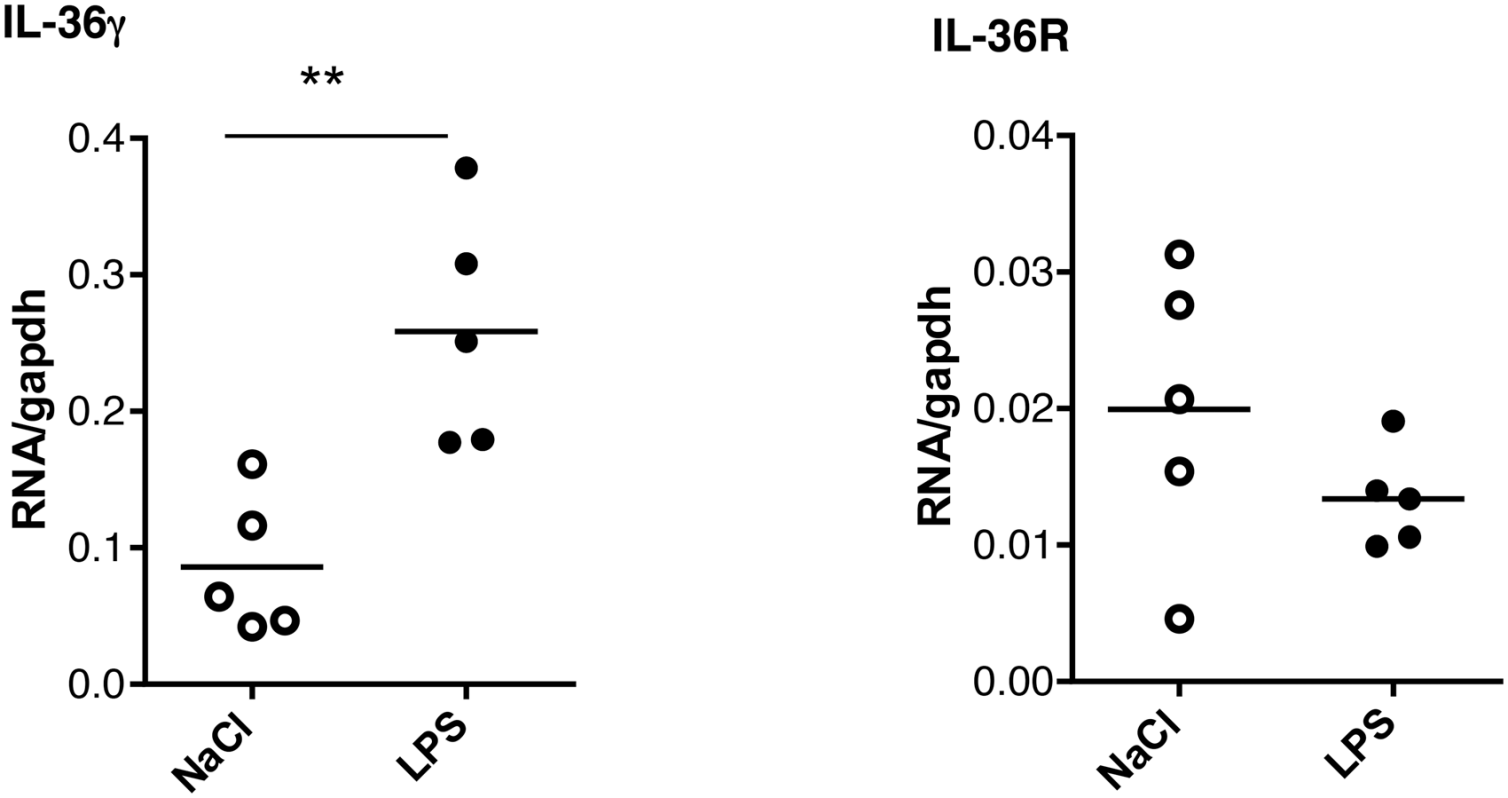
Elevated lung IL-36γ mRNA levels following LPS instillation. Lung total RNA was extracted 4 h after intra-tracheal instillation of LPS (n = 5 mice) or saline (n = 5 mice). The results represent the levels of *Il36g* or *Il36r* mRNA determined by RT-qPCR normalized to *Gapdh* mRNA. **P<0.01, as assessed by Mann-Whitney test.

**Fig 2:**
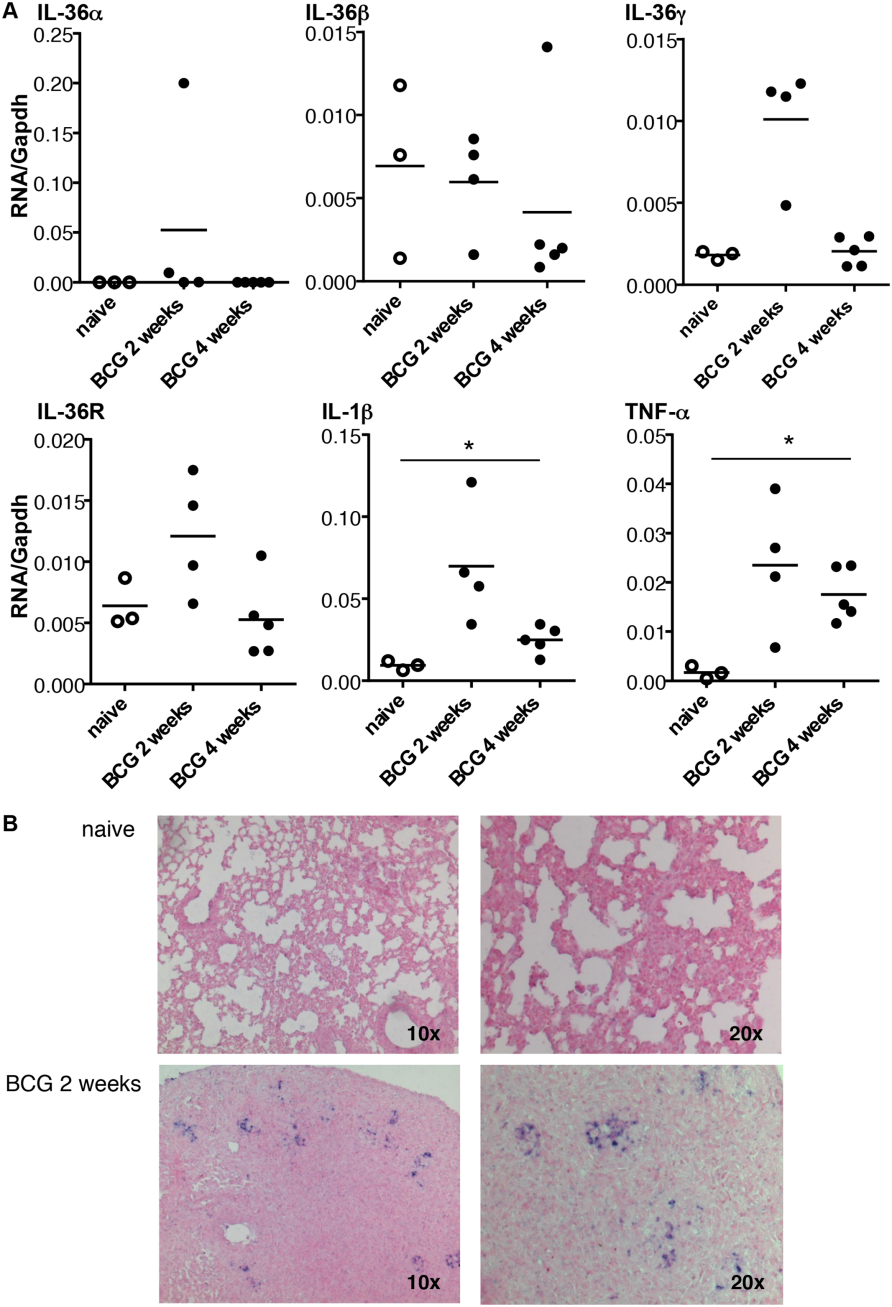
Kinetic of IL-36 cytokines, IL-36R, IL-1β and TNF-α mRNA expression after *M*. *bovis* BCG infection. mRNA levels of *Il36a*, *Il36b*, *Il36g*, *Il36r*, *Il1b*, and *Tnfa* measured by RT-qPCR and normalized to *Gapdh* mRNA levels from the lungs of C57BL/6 wild-type mice 2 weeks (n = 4) and 4 weeks (n = 5) after *M*. *bovis* BCG infection. Three naïve mice were included as controls (A). *In situ hybridization* was performed using a digoxigenin-labeled riboprobe complementary to *Il36g* mRNA (B). The micrographs are representative of lungs from one naïve mice (upper panel) and one infected mice 2 weeks after *M*. *bovis* BCG inoculation (lower panel). *P<0.05, as assessed by Kruskal-Wallis, followed by Mann-Whitney test.

To determine how IL-36 cytokines are regulated during lung inflammation, mRNA expression of IL-36 cytokines and IL-36R was first measured 6 h after intratracheal instillation of LPS. There was a significant increase in pulmonary levels of IL-36γ mRNA that remained elevated at 24 hours in LPS-treated mice as compared to controls instillated with saline (Fig 1). In contrast, IL-36α, IL-36β and IL-36R expression was either unchanged or remained undetectable after LPS exposure (data not shown). IL-36 cytokine and IL-36R mRNA expression was next determined after intravenous inoculation of *M*. *bovis* BCG infection (5x10^6^ CFU living *M*. *bovis* BCG Pasteur strain 1173 P2). IL-36γ mRNA levels were transiently increased 2 weeks after infection (P = 0.057), and IL-36g mRNA was present in lung granulomatous lesions after *M*. *bovis* BCG infection, as shown by *in situ* hybridization (Fig 2A and 2B). Of note, *M*. *bovis* BCG infection barely affected pulmonary IL-36α, IL-36β and IL-36R mRNA levels, although IL-1β and TNF-α expression was increased (Fig 2A). Thus, lung inflammation was associated with an upregulation of inflammatory cytokines such as TNF and IL-1 that included IL-36γ.

### IL-36R is dispensable for the control of M. bovis BCG infection

**Fig 3:**
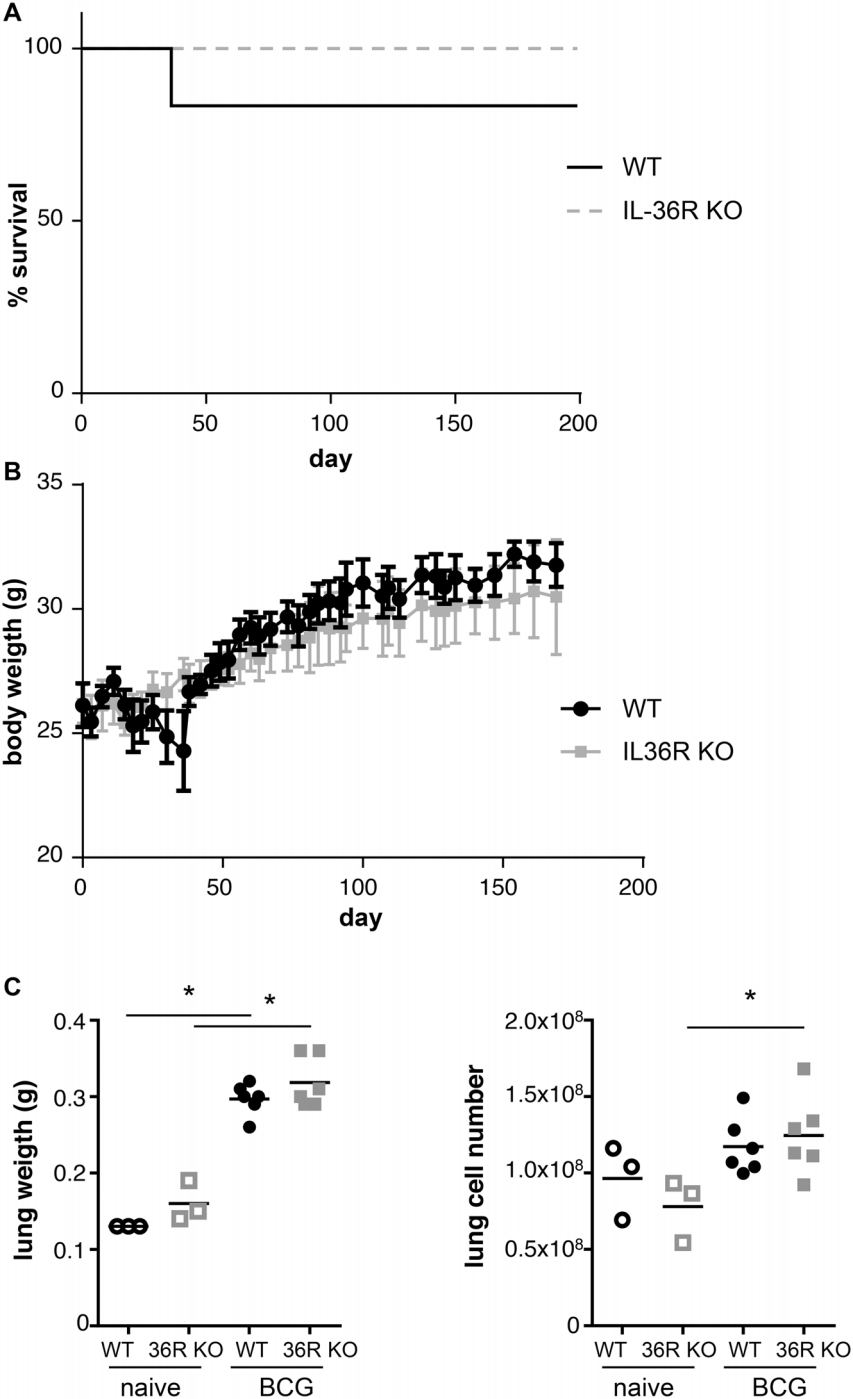
Role of IL-36R signaling in *M*. *bovis* BCG infection. Wild-type and IL-36R deficient mice were inoculated intravenously with *M*. *bovis* BCG (n = 12 per group). Six mice from each genotype were sacrificed at 28 days and the other mice were followed for up to 180 days. The percentage of survival (A) and evolution of body weight (B) for WT and IL-36R deficient mice are depicted. (C) Total lung weight and absolute lung cell numbers at 28 days after infection are represented in left and right panels, respectively. No significant differences were observed between WT and IL-36R KO mice, as assessed by log-rank (Mantel-Cox) test (A) or two-way ANOVA for repeated measures, followed by Sidak’s multiple comparison test (B). *P<0.05, as assessed by Kruskal-Wallis, followed by Mann-Whitney test (C). Black and grey symbols represent WT and IL-36R deficient mice, respectively.

**Fig 4:**
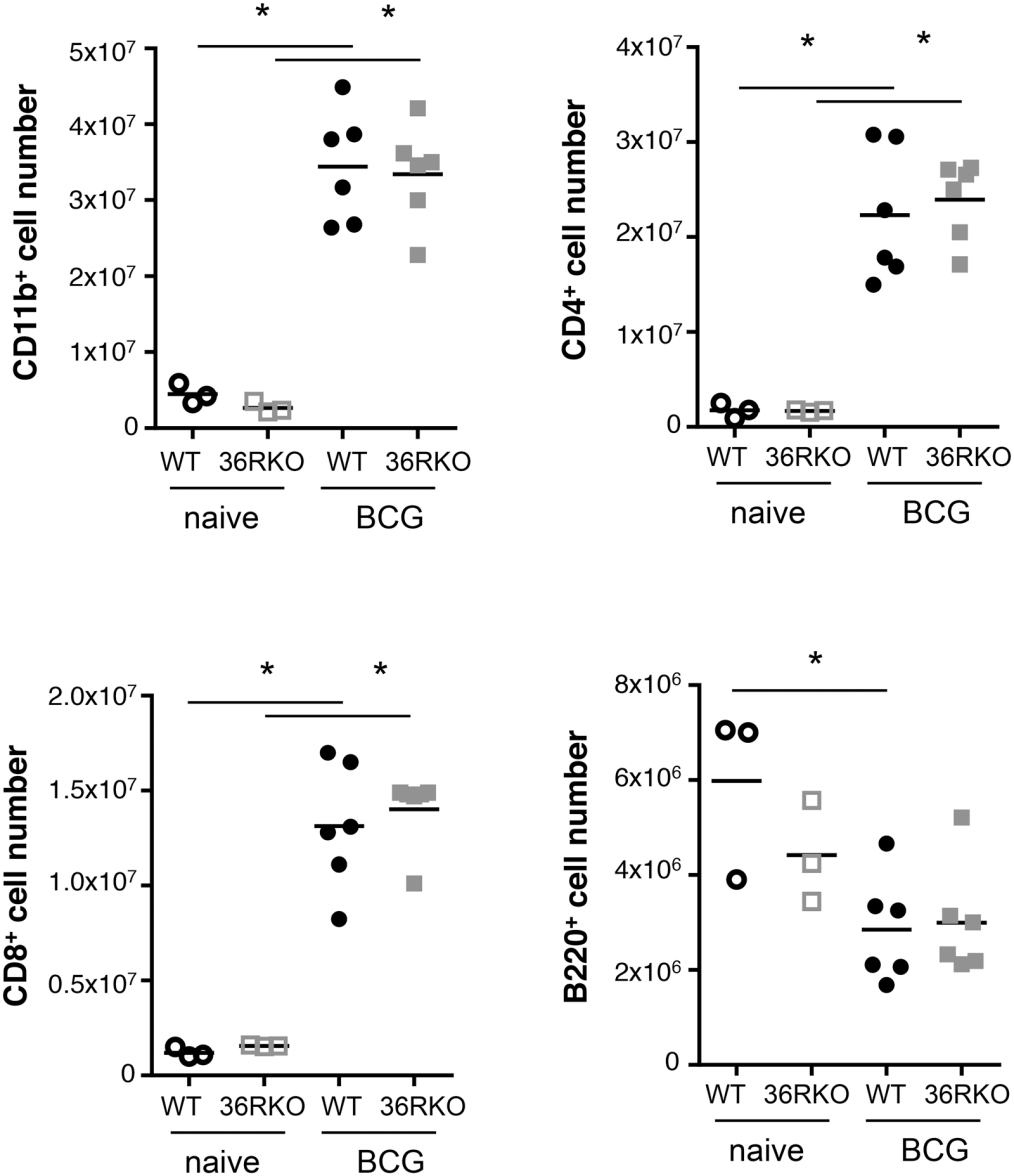
Changes in lung cell populations following *M*. *bovis* BCG inoculation. Lung cells were isolated from 3 naive WT and IL-36R deficient mice, and from 6 WT and 6 IL-36R deficient mice 4 weeks after intravenous *M*. *bovis* BCG inoculation. The results represent CD11b^+^, CD4^+^, CD8^+^, and B220^+^ cell numbers as assessed by flow cytometric analysis. *P<0.05, as assessed by Kruskal-Wallis, followed by Mann-Whitney test.

**Fig 5:**
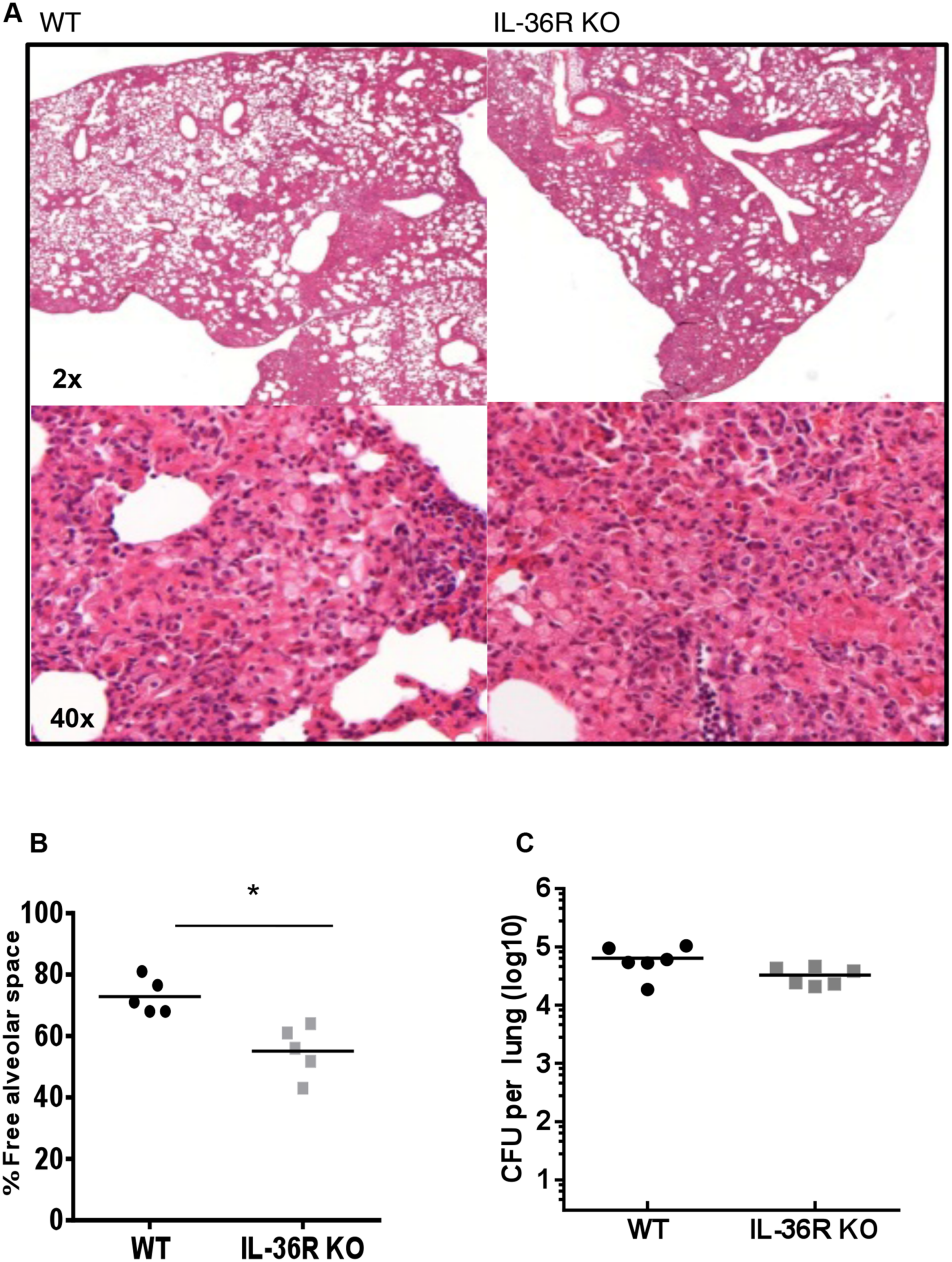
Histopathology and bacterial loads in wild-type and IL-36R deficient mice. Hematoxylin eosin staining of lung sections 4 weeks after *M*. *bovis* BCG infection of WT (left panels) and IL-36R deficient (right panels) mice (original magnifications X2, upper panel, and X40, lower panel) (A). Determination of free alveolar space on lung sections. Data are represented as the mean ± SD of percentage of lung lesion free/total lung tissue in 5 mice per group with 1 or 2 lobes analyzed per mouse (B). Lung bacterial loads at 4 weeks post-infection are represented as CFU per lung (n = 6 mice per group) (C). **P<0.01 as assessed by Mann Whitney test.

**Fig 6:**
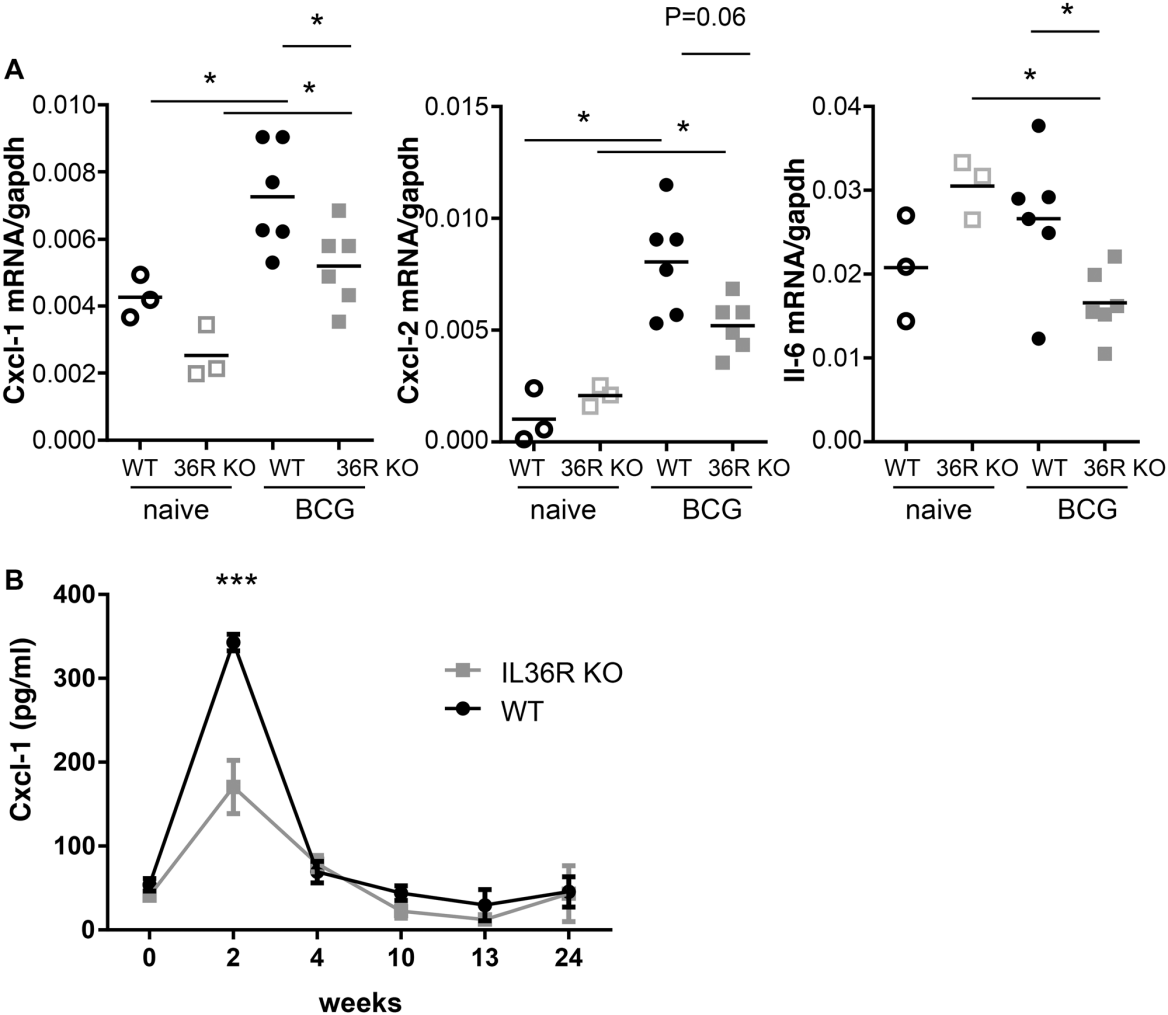
CXCL1 expression in response to *M*. *bovis* infection in IL-36R deficient and wild-type mice. **A.** Total RNA was prepared from the lungs of naïve (open symbols) and *M*. *bovis* BCG infected (closed symbols) WT (black circles) and IL-36R deficient (grey squares) mice. The results represent *Cxcl1*, *Cxcl2*, *and Il6* mRNA levels determined by RT-qPCR normalized to *Gapdh* mRNA levels in individual mice. *P<0.05, as assessed by Kruskal-Wallis, followed by Mann-Whitney test. **B.** Serum levels of Cxcl1 were measured by ELISA in WT (black symbols) and IL-36R deficient (grey symbols) mice. The results represent means ± SEM of 6 mice per group. Two-way ANOVA for repeated measures, followed by Sidak’s multiple comparison test was used to compare IL-36R deficient and WT mice. ***P<0.001 only at week 2.

Previous results showed that IL-36R signaling is involved in Th1 responses following *M*. *bovis* BCG infection [5]. Since IL-36γ expression is transiently upregulated and IL-36γ is present in lung granulomas associated with *M*. *bovis* BCG infection, we thus examined whether IL-36R signaling is involved in host responses against *M*. *bovis* BCG infection. IL-36R KO and WT mice were inoculated with *M*. *bovis* BCG (5 x 10^6^ CFU per mouse, i.v.). Survival and body weight were not significantly different in WT and IL-36R deficient mice up to 6 months post infection (Fig 3A and 3B). Lung inflammatory responses and bacterial clearance were examined at day 28 post *M*. *bovis* BCG injection. Lung weight was increased in *M*. *bovis* BCG infected mice as compared to naïve mice, but there was no difference between WT and IL-36R deficient mice (Fig 3C). Total lung infiltrating inflammatory cell numbers were also enhanced after *M*. *bovis* BCG infection, the difference with naïve mice reaching statistical significance in IL-36R KO mice (Fig 3C). Absolute numbers of lung infiltrating CD11b^+^ myeloid cells, CD4^+^ and CD8^+^ T cells were also enhanced following *M*. *bovis* BCG infection (Fig 4), but there was no significant difference between WT and IL-36R deficient mice. B220 cell numbers were decreased with the same magnitude in the two mouse strains in response to *M*. *bovis* BCG infection. There was a significant decrease in free alveolar space in the lungs of IL-36R deficient mice as compared to WT mice (Fig 5A and 5B), as previously observed [5], suggesting the presence of enhanced inflammatory responses in the lungs of IL-36R KO mice. However, the pulmonary bacterial burdens were not significantly different in IL-36R KO and WT mice (Fig 5C). Furthermore, at 6 months post-infection, the histological findings were no longer different in IL-36R deficient and WT mice and the presence of *M*. *bovis* BCG was undetectable by CFU and Ziehl-Neelsen staining in both mouse strains (data not shown). Transcript levels of TNF-α, IL-6, IL-10, IL-12p40, CXCL1, CXCL2, IFN-γ, GM-CSF, G-CSF, iNOS, T-bet, GATA3, RORγt were measured in the lungs at day 28 post *M*. *bovis* BCG infection. The mRNA levels were not different between IL-36R deficient and WT mice, with the exception of CXCL1, CXCL2, and IL-6 that were lower in IL-36R KO than in WT mice (Fig 6A). The serum levels of CXCL1 that peaked 2 weeks post-*M*. *bovis* BCG infection were significantly lower in IL-36R KO than in WT mice at this time-point (Fig 6B). Thus, the transiently increased lung inflammation in *M*. *bovis* BCG infected IL-36R deficient mice did not translate in an increase in inflammatory cytokine expression, and was associated with a decrease in CXCL1 expression.

### Limited contribution of IL-36R, compared to IL-1RI and TNF-α pathways in the control of M. tuberculosis infection

**Fig 7:**
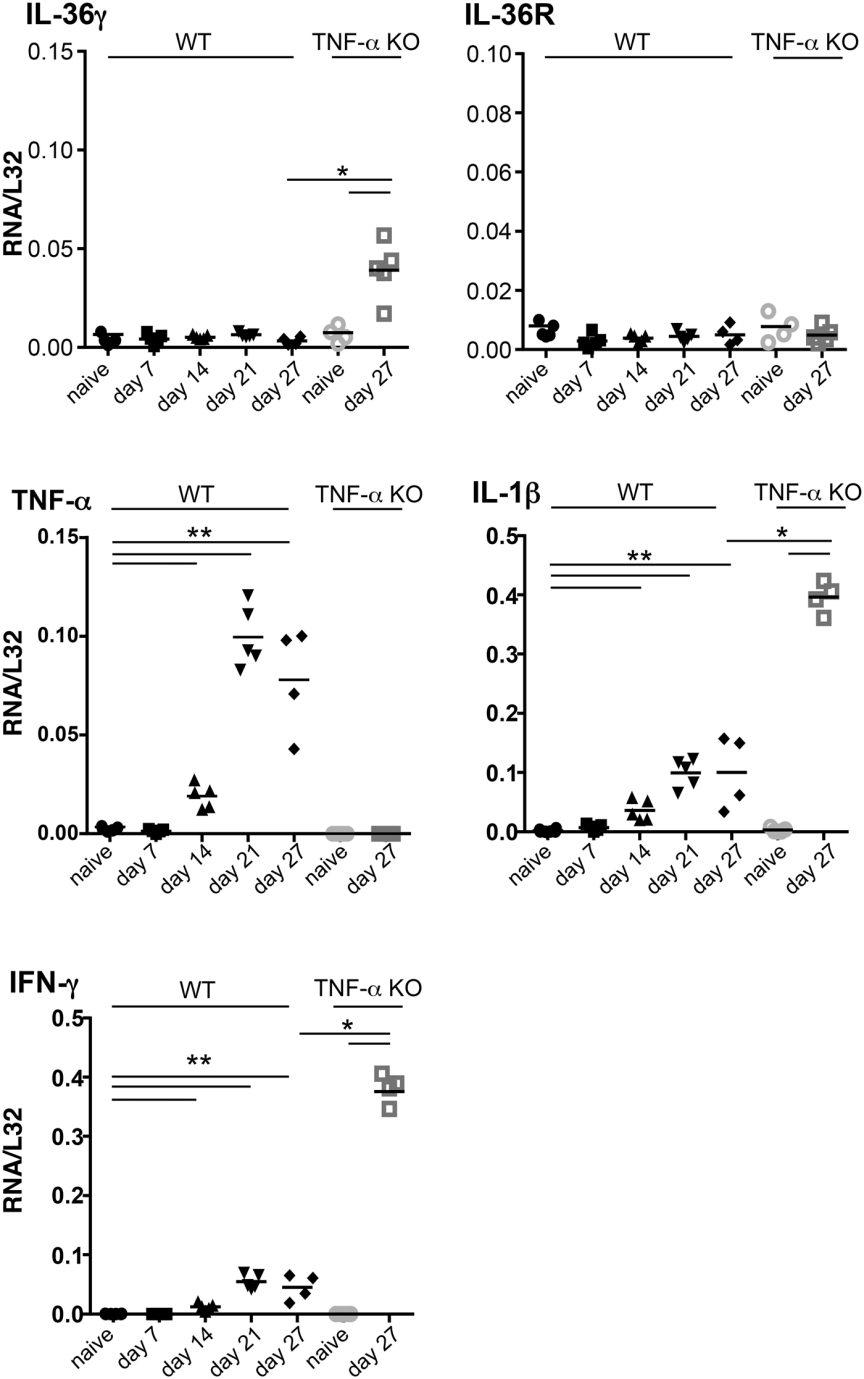
Kinetics of pulmonary cytokine expression after *M*. *tuberculosis* infection. Total RNA was prepared from the lungs of naïve WT (black symbols) and TNF-α deficient mice (grey symbols) and at days 7, 14, 21 and 27 after *M*. *tuberculosis* infection in WT (black symbols) and at day 27 in TNF-α deficient mice (grey symbols). The results represent the levels of *Il36g*, *Il36r*, *Tnfa*, *Il1b*, and *Ifng* mRNA determined by RT-qPCR normalized to *L32* mRNA (n = 5 per group). *P<0.05, **P<0.01, as assessed by Kruskal-Wallis, followed by Mann-Whitney test (C).

The expression levels of IL-36α, β, γ, IL-1β, TNF-α and IFN-γ mRNA were next determined in the lungs of WT and TNF-α deficient mice at different time-points following aerogenic infection with *M*. *tuberculosis*. IL-36γ and IL-36R mRNA levels remained unchanged up to 27 days after *M*. *tuberculosis* infection in WT mice. In contrast, the transcript levels of IL-1β, TNF-α, and IFN-γ were significantly increased at day 14 and were further upregulated at day 21 and 28 (Fig 7). In the highly susceptible, *M*. *tuberculosis* infected TNF-α deficient mice, the mRNA levels of IL-1β and IFN-γ were even higher than in WT mice. Furthermore, there was a significant increase of IL-36γ mRNA levels in TNF-α deficient mice infected with *M*. *tuberculosis*.

**Fig 8:**
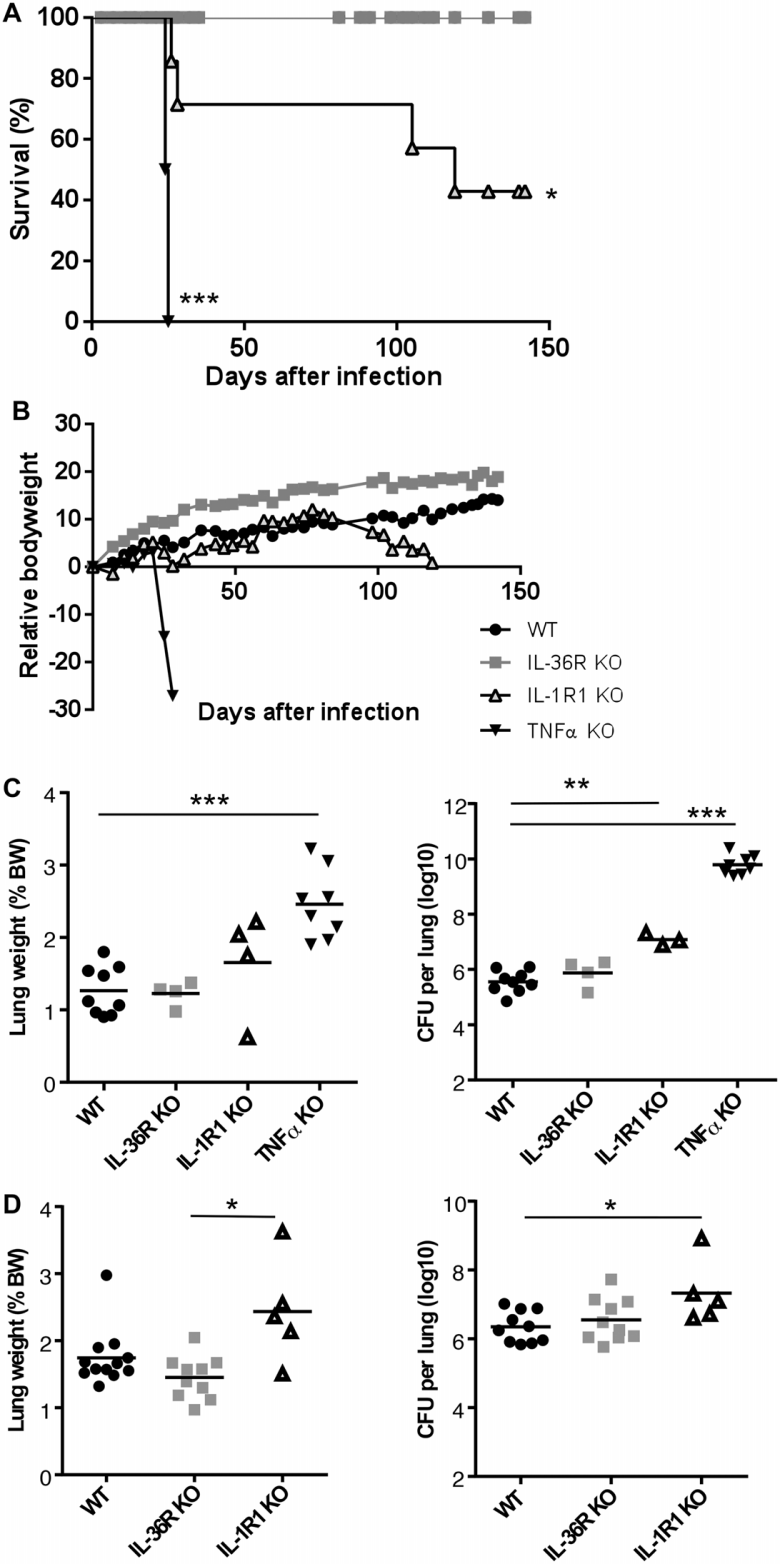
IL-36R is dispensable to control acute *M*. *tuberculosis* infection. Mice deficient for IL-36R, IL-1R1 or TNF-α, and WT mice were exposed to an aerogenic dose of *M*. *tuberculosis* H37Rv and monitored for survival (A) and body weight. The results represent mean body weight ± SEM values of 8–14 mice per group, pooled from 2 independent experiments. Lung weight (left panels) and bacterial loads (right panels) were determined in TNF-α deficient, IL-36R deficient, IL-1R1 deficient, and WT mice at 1 month (C) and 4 months (D) post infection. Results are expressed as individual values and mean (n = 4–9 for lung weight, n = 3–9 for CFU) from 1 experiment at 1 month and (n = 5–12 for lung weight, n = 5–10 for CFU) from 2 experiments at 4 months. *P<0.05, **P<0.01, ***P<0.001 as compared to the wild-type controls (assessed by Kruskal-Wallis, followed by Mann-Whitney test).

**Fig 9:**
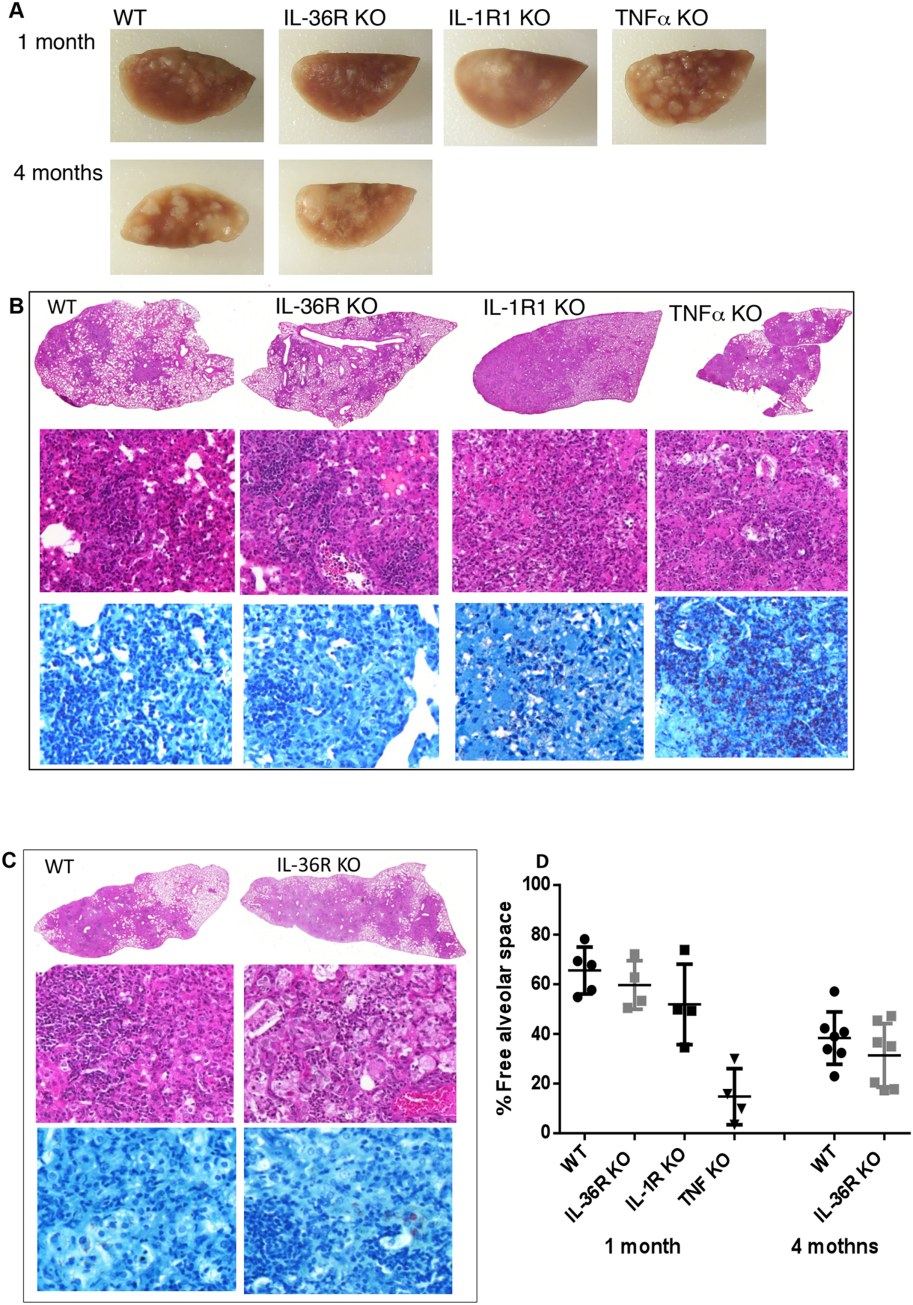
Controlled lung pathology after *M*. *tuberculosis* infection in the absence of IL-36R pathway. Macroscopic lung lesions of IL-36R deficient, IL-1R1 deficient, TNF-α deficient, and WT mice at 1 month, and of IL-36R deficient and WT mice at 4 months post *M*. *tuberculosis* infection (A). Histological studies were performed at 1 month in IL-36R deficient, IL-1R1 deficient, TNF-α deficient, and WT mice (B), and at 4 months in IL-36R deficient and WT mice (C). Representative lung H&E (1x and 40x magnifications) and Ziehl-Neelsen (60x magnification) staining at 1 and 4 months post *M*. *tuberculosis* infection. Free alveolar space quantification is represented as the mean ± SD of percentage lung lesion free/total lung tissue in 4–7 mice per group with 1–2 lobes analyzed per mouse (D). Statistical analysis was one-way ANOVA, no statistically significantly differences were observed between animal groups.

**Fig 10:**
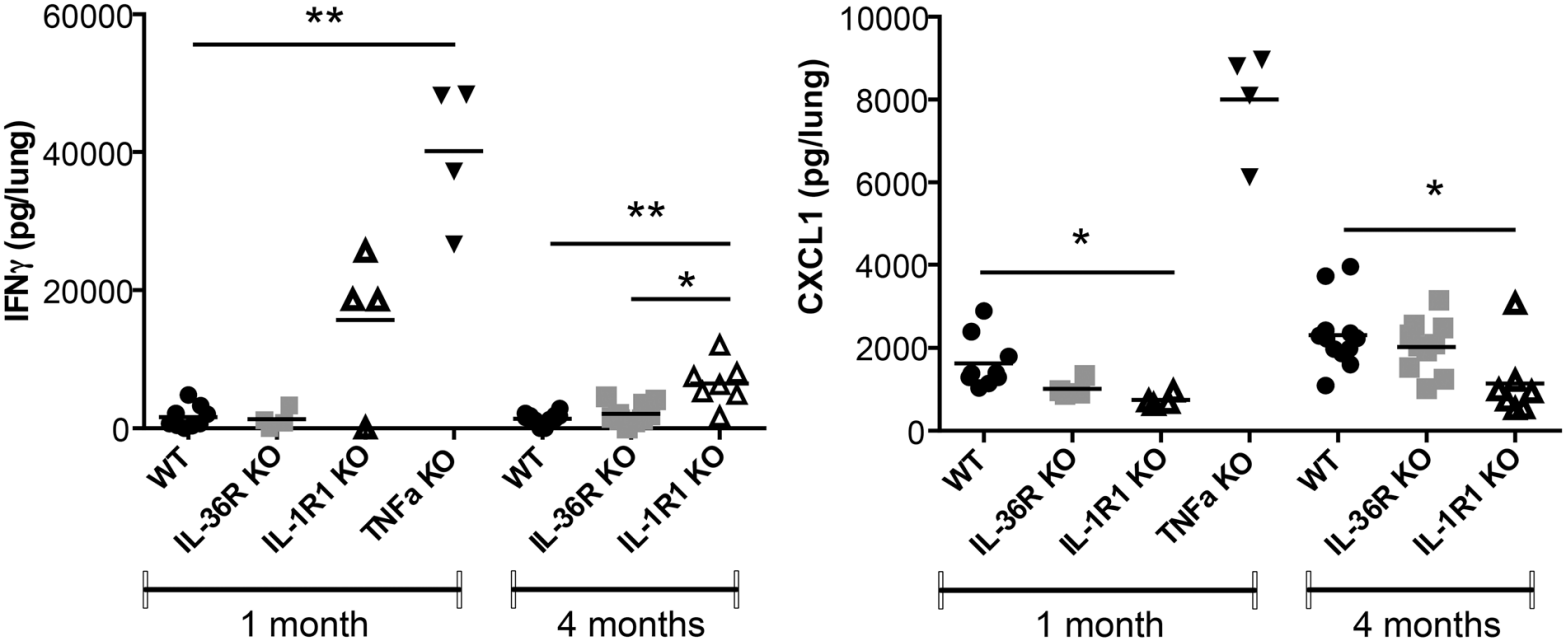
Lung IFN-γ and Cxcl-1 levels in *M*. *tuberculosis* infection. The levels of IFN-γ and Cxcl-1 protein were determined in lung homogenates 1 month post aerogenic *M*. *tuberculosis* infection in TNF-α deficient, IL-36R deficient, IL-1R1 deficient, and wild-type mice, and 4 months post-infection in IL-36R deficient and WT mice. Results are expressed as mean +/- SEM of n = 3–9 from 1 experiment at 1 month, n = 5–10 from 2 experiments at 4 months. P<0.05 *; P<0.01 ** as compared to the WT mice.

**Fig 11:**
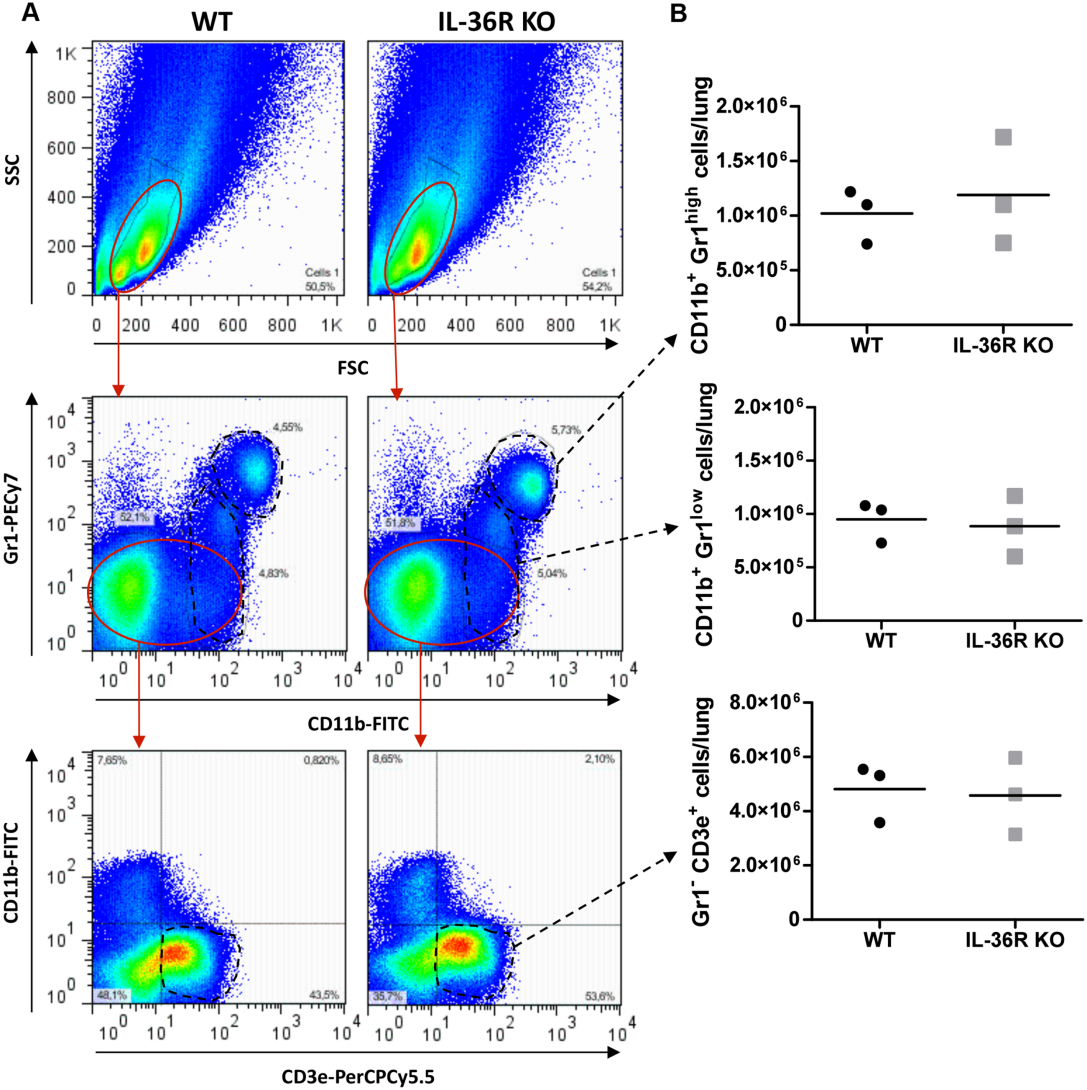
Unaffected lung inflammatory cell infiltration in the absence of IL-36R pathway after *M*. *tuberculosis* infection. Infiltrating cells in the lungs of IL-36R deficient (right panels) and wild-type mice (left panels) were isolated on day 28 after *M*. *tuberculosis* H37Rv infection, and analyzed by flow cytometry for the expression of CD11b, Gr1 (Ly6G/6C) and CD3. Gating strategy and representative dot blots are shown (A). Results expressed as the absolute number of CD11b^+^Gr1^high^, CD11b^+^Gr1^low^ and Gr1^low^ CD3ε^+^ cells per lung are from 3 individual mice with mean values as horizontal bars (B).

To assess the contribution of the IL-36 pathway to host responses against *M*. *tuberculosis* infection, WT and IL-36R deficient mice were infected by intra-nasal instillation of *M*. *tuberculosis*. Mice deficient for IL-1RI and TNF-α were included as controls since both mouse strains have previously been reported to exhibit a reduced control of acute *M*. *tuberculosis* infection associated with enhanced lethality. IL-36R deficient mice had a similar evolution of body weight and survival as compared to WT mice up to 4 months after *M*. *tuberculosis* infection. In contrast, 100% of TNF-α mice died or had to be sacrificed within one month post infection and IL-1R1 deficient mice exhibited an intermediate susceptibility with a 50% survival during the 4 months follow-up period (P = 0.0455) (Fig 8A and 8B). Consistent with these findings, lung weights and bacterial loads were significantly increased in TNF-α deficient mice as compared to WT mice (P<0.0001) (Fig 8C). In addition, macroscopic and histological examination showed that the lungs of TNF-a mice exhibited massive inflammatory responses after one month of infection with reduced free alveolar space (Fig 9) and enhanced bacterial load as assessed by Ziehl-Neelsen staining (Fig 9). Bacterial burden was significantly increased in IL-1R1 deficient mice as compared to WT mice at 1 month (P = 0.0091) and 4 months (P = 0.0193) post-infection (Fig 8C and 8D). There was no difference regarding lung weight, bacterial load or histopathological findings between IL-36 KO and WT mice at 1 or 4 months post-infection (Figs 8 and 9). Lung inflammatory response was further analyzed by assessing the level of the typical pro-inflammatory, Th1 cytokine IFN-γ and of the chemokine CXCL1 (Fig 10). In line with their increased lung inflammation, lung IFN-γ levels of were significantly increased in TNF-α deficient mice at 1 month post infection as compared to WT mice, as well as in IL-1RI deficient as compared to WT mice at 4 months, while IL-36R deficient mice exhibited IFN-γ levels similar to WT controls. The levels of CXCL1 were increased in TNF-α deficient mice, although this did not reach significance, but were significantly decreased in IL-1RI deficient mice at 1 and 4 months post infection. There was no difference in the lung levels of CXCL1 in IL-36R deficient and WT mice at 1 and 4 months post-infection. Finally, flow cytometric analysis of lung infiltrating cells showed similar numbers of granulocytic and monocytic CD11b^+^ Gr1^+^ and CD11b^+^ Gr1^-^ cells in IL-36R deficient and WT mice, as well as similar numbers of CD3^+^ T cells at 1 month after infection (Fig 11). Therefore, IL-36R deficiency did not impair the control of *M*. *tuberculosis* infection as did the absence of TNF-α or IL-1R1 signalling.

## Discussion

We show here that IL-36γ mRNA expression is enhanced during acute lung inflammation induced by intratracheal LPS instillation or *M*. *bovis* BCG systemic infection in WT mice and after aerogenic *M*. *tuberculosis* infection of highly susceptible, TNF-α deficient mice. To understand whether IL-36γ expression represents a host defense mechanism essential to fight the infection, or is rather a consequence of the enhanced inflammatory response induced by the uncontrolled infection, we addressed the relevance of IL-36 pathway in controlling lung inflammation and infection by mycobacteria. IL-36R deficient mice had transiently increased lung pathology and reduced lung chemokine CXCL1 production following *M*. *bovis* BCG infection, but without long term consequences on bacterial control. Indeed, while TNF-α KO mice succumbed after acute *M*. *tuberculosis* infection with high bacterial loads and severe inflammatory responses and IL-1R1 KO mice exhibited an intermediate phenotype, the survival, bacterial clearance, and inflammatory responses were similar in IL-36R deficient and WT mice. Taken together, these results indicate that IL-36R signaling has limited contribution on host responses to mycobacterial infections.

Like other IL-1 family members, IL-36 is produced by stimulated innate immune cells, including macrophages and DC, and may thus contribute to host defense against pathogens. Human peripheral blood mononuclear cells produce IL-36γ and IL-36Ra in response to *Aspergillus fumigatus* infection, and the addition of IL-36Ra inhibits the production of IL-17 and IFN-γ, suggesting that IL-36 is involved in the production of these cytokines by PBMC in response to *Aspergillus fumigatus* infection [32]. Human monocyte-derived DC (MDC) and polarized M1 macrophages produce IL-36γ in response to IL-1, TNF-α, and IFN-γ, an expression dependent on t-bet, the transcription factor T-box expressed in T cells [33]. Mouse bone marrow-derived DC (BMDC) express IL-36γ and IL-36α either constitutively or in response to various stimulations [2]. Consistent with these *in vitro* findings we show here the presence of IL-36γ in granulomatous lung lesions from *M*. *bovis* BCG infected mice.

IL-36R is expressed by BMDC and IL-36 stimulates the production of several cytokines and chemokines, as well as the expression of MHC class 2 molecules and co-stimulatory receptors [2]. Similarly, IL-36 stimulates the expression of cytokines and induces MDC maturation with enhanced MHC class 2, CD83 and CD86 expression [3], [4]. IL-36R is also expressed by CD4+ T cells, in particular naïve CD4+ T cells, and IL-36 induces Th1 responses *in vitro* and *in vivo* [5]. The stimulatory effects of IL-36 on Th1 polarization are dependent on the release of IL-12 by DC and the production of IL-2 by CD4+ T cells, which in turn induces IL-12Rβ2 expression [5]. These findings suggested that IL-36 could play an important role in host defense against mycobacterial infections. However, we show here that host responses involved in *M*. *bovis* BCG and *M*. *tuberculosis* infection containment and survival were largely independent of IL-36R signaling. Consistent with these results, IL-36R signaling is also dispensable for the control of footpad infection with *Leishmania guyanensis*, another infectious model in which Th1 responses play a critical role for parasitic clearance (Lamacchia, Gabay et al, unpublished data).

Recent data indicate that various inflammatory stimulants also induce IL-36 production by bronchial epithelial cells, including TNF-α, IL-1β and IL-17, as well as cigarette smoke condensate [34], [35]. Production of IL-36γ by bronchial epithelial cells is also markedly stimulated by infectious agents, including *Pseudomonas aeruginosa* [36] and rhinovirus, with higher rhinovirus-induced IL-36γ in epithelial cells from asthmatic patients [37]. In *M*. *bovis* BCG infected mice the levels of CXCL1, CXCL2, and IL-6 were transiently lower in IL-36R KO as compared to WT mice, with no consequence on the severity of lung infection and survival. Similarly, there was no difference in lung inflammatory responses following aerogenic *M*. *tuberculosis* infection in IL-36R KO and WT mice. Furthermore, although IL-36γ production was enhanced in response to intratracheal LPS instillation, the severity of inflammatory responses, as assessed by cellular composition and cytokine levels in bronchoalveolar lavages, was unaffected by IL-36R deficiency (Woldt, Gabay et al, unpublished results).

Regarding other IL-1 family cytokine pathways, mice deficient in IL-1α/β and IL-1R1 signaling exhibit enhanced bacterial loads and reduced survival in response to *M*. *tuberculosis* infection, although adaptive responses are essentially unaltered [38], [20], [23], [24]. A mechanism through which IL-1 may control *M*. *tuberculosis* infection has recently been proposed, namely IL-1 induced release of prostaglandin E2, which exerts an inhibitory effect on type I IFN production, leading to better bacterial containment [39]. In our study, the phenotype of IL-1R1 KO mice was less severe than in TNF-α deficient mice. This finding is in accordance with the predominant role of TNF-α in the control of latent and primary tuberculosis infection in humans [13], [14], [15], [16], and mice [24].

Several data obtained in mice and humans indicate that IL-36R signaling is involved in the pathogenesis of psoriasis. Transgenic mice overexpressing IL-36α exhibited transient skin changes reminiscent of psoriasis. This phenotype was abrogated when transgenic mice were crossed with IL-36R or IL-1RAcP deficient mice, but strongly exacerbated when IL-36α transgenic mice were crossed with IL-36Ra deficient mice [26]. Treatment of SCID mice engrafted with human psoriasis skin with a monoclonal anti-human IL-36R neutralizing antibody led to substantial normalization of engrafted skin pathology [40]. Imiquimod, a TLR7 agonist, can induce psoriasis-like lesions in human and mice. This effect is almost completely dependent upon IL-36R signaling [41]. Finally one of the most compelling evidence regarding a role of IL-36R signaling in psoriasis is the presence of a variety of IL36RN gene mutations leading to non-functional IL-36Ra in some patients with generalized pustular psoriasis (reviewed in [25]).

In the search of new therapeutic strategies limiting the risk of tuberculosis reactivation, our previous studies showed that targeting selectively soluble TNF but sparing membrane TNF by Dominant Negative-TNF inhibitor molecules did not compromise immune responses to both *M*. *bovis* BCG and *M*. *tuberculosis* infections in contrast to other TNF neutralizing molecules [42], while efficiently protecting mice against acute inflammatory reactions [30]. Here we show that the host response to *M*. *tuberculosis* was preserved in the complete absence of IL-36R signaling. Our data suggest that targeting IL-36R signaling, for example in the context of generalized pustular psoriasis or other forms of psoriasis [43] may be considered as a new, safe therapeutic strategy unlikely to compromise host immunity to mycobacteria, thereby reducing the risk of tuberculosis reactivation that is associated with TNF antagonists.

In conclusion, despite *in vitro* and *in vivo* findings indicating that IL-36R signaling stimulates Th1 responses, and that IL-36γ is over-expressed in the inflamed lung, our results show that the IL-36 pathway is dispensable for the control of mycobacterial infection. This suggests that neutralizing IL-36 may allow therapeutic interventions for severe inflammatory indications while sparing host control of tuberculosis.
